# Insights into the Sensing Mechanism of a Metal-Oxide Solid Solution via *Operando* Diffuse Reflectance Infrared Fourier Transform Spectroscopy

**DOI:** 10.3390/nano13192708

**Published:** 2023-10-05

**Authors:** Elena Spagnoli, Matteo Valt, Andrea Gaiardo, Barbara Fabbri, Vincenzo Guidi

**Affiliations:** 1 Department of Physics and Earth Sciences, University of Ferrara, Via Saragat 1/C, 44122 Ferrara, Italy; barbara.fabbri@unife.it; 2 MNF-Micro Nano Facility, Sensors and Devices Center, Bruno Kessler Foundation, Via Sommarive 18, 38123 Trento, Italy; mvalt@fbk.eu (M.V.); gaiardo@fbk.eu (A.G.)

**Keywords:** DRIFT spectroscopy, metal-oxide solid solution, chemoresistive gas sensors

## Abstract

Recently, the influence of Nb addition in the oxide solid solution of Sn and Ti was investigated with regard to the morphological, structural and electrical properties for the production of chemoresistive gas sensors. (Sn,Ti,Nb)_x_O_2_-based sensors showed promising features for ethanol monitoring in commercial or industrial settings characterized by frequent variation in relative humidity. Indeed, the three-metal solid solution highlighted a higher response level vs. ethanol than the most widely used SnO_2_ and a remarkably low effect of relative humidity on the film resistance. Nevertheless, lack of knowledge still persists on the mechanisms of gas reaction occurring at the surface of these nanostructures. In this work, *operando* Diffuse Reflectance Infrared Fourier Transform spectroscopy was used on SnO_2_- and on (Sn,Ti,Nb)_x_O_2_-based sensors to combine the investigations on the transduction function, i.e., the read-out of the device activity, with the investigations on the receptor function, i.e., compositional characterization of the active sensing element in real time and under operating conditions. The sensors performance was explained by probing the interaction of H_2_O and ethanol molecules with the material surface sites. This information is fundamental for fine-tuning of material characteristics for any specific gas sensing applications.

## 1. Introduction

Chemoresistive gas sensors belong to the class of chemical sensors, and typically consist of a sensitive semiconducting layer deposited on the top of a substrate equipped with electrodes used to measure the electrical signal, which strongly depends on the gas composition of the surrounding environment. The sensitive film is typically thermally activated using a built-in heater. This class of sensors has been extensively researched since the late 1970s due to the low cost, flexibility of production, ease of use, long-term stability and large number of detectable gases. Indeed, their initial successful commercialization as alarm devices has increased the interest in industries for their use in new applications [1], and in the scientific community for the search of innovative materials to meet the market demands in terms of sensitive, selective and stable gas sensors.

Along with the efforts to develop customized devices to match the application demands, the research also focused on the implementation of experimental investigations to fill the lack of knowledge on the mechanisms of gas reaction occurring at the surface of the nanostructures. Indeed, this information is fundamental for fine-tuning of material characteristics for any specific gas sensing applications [2,3].

To this aim, Diffuse Reflectance Infrared Fourier Transform (DRIFT) spectroscopy is one of the most advanced and effective methods to examine the composition of rough-surfaced solid samples [4]. For this reason, the first work on *operando* studies of the gas sensing mechanism, published in 1995, combined DRIFT characterizations with simultaneous resistance measurements on a CdGeON sensor [3,5]. In the following years, the electrodes–heater configuration was optimized to lower the energy consumption and miniaturize the device, making *operando* characterizations more challenging. In 2013, Barsan et al. investigated, with *operando* DRIFT spectroscopy, the reaction mechanism occurring on SnO_2_, the most employed material for the production of chemoresistive sensors [1], in the presence of water vapor, CO and H_2_ [6]. It was demonstrated that water and reducing gases shared the same reaction sites, namely preadsorbed oxygen ions ($O_{ads}^{-})$ [6], and that competitive reactions negatively affected the sensor performance in the presence of humidity. Indeed, the effect of relative humidity (RH%) on the baseline and on the sensitivity often reduces sensor accuracy and complicates calibrations in real working conditions [7].

Among the wide palette of applications, gas sensors play an important role in ensuring ethanol levels within safe and acceptable limits because it is highly flammable, and it poses serious health risks to people working in commercial or industrial settings. Nevertheless, monitoring of ethanol with a chemoresistive gas sensor in ethanol-producing facilities, such as breweries, distilleries, wineries [8] and manufacturing of chemicals, [9] can be complicated because its content variation in the air is frequently correlated with inadequate ventilation, which also influences the relative humidity in the confined areas. Despite a significant amount of literature confirming high sensing capabilities vs. ethanol detection achieved with structural engineering of metal-oxide films, only few works demonstrated a low effect of the relative humidity on the response level or on the film resistance. Table S1 summarizes some relevant studies on an optimized semiconducting metal-oxide (SMOX) gas sensors for ethanol detection, displaying, when data were available from the reference, the response% decrease at 60 RH% oxides [7,10,11,12,13,14,15,16,17,18,19,20,21,22]. Promising results were obtained in 2009 by Tricoli et al. in terms of low cross-sensitivity to humidity during ethanol detection with sensors based on a solid solution of Sn and Ti [10]. However, the (Sn,Ti)_x_O_2_ solid solution presented some drawbacks such as the growth of the film resistance with the increase in the Ti concentration, grain coalescence at temperatures necessary for the formation of the solid solutions and anatase-to-rutile phase transition [10]. Previous investigations have shown that doping with transition metals can inhibit particle growth and improve thermal stability. In [23], Ferroni et al. investigated the effect of Nb^5+^ incorporation within a TiO_2_ lattice, proving that it can inhibit grain growth and anatase-to-rutile transition. Recently, the influence of Nb addition in the solid solution of Sn and Ti was investigated with regard to the morphological, structural and electrical properties [7,24]. Firstly, addition of a low concentration of Nb (e.g., 1.5% of the total metal amount) increased the conductance of the film based on (Sn,Ti)_x_O_2_. Secondly, Nb avoided grain coalescence well above the operating temperature of the films. Finally, it enhanced the surface reactivity with creation of cation vacancies and trivalent cations (Ti^3+^), which improved the sensitivity to some gases, such as ethanol. Moreover, the highly desired low humidity effect of (Sn,Ti)_x_O_2_ was maintained also for (Sn,Ti,Nb)_x_O_2_ [7].

Considering the useful receptor characteristics of the metal-oxide solid solution based on Sn, Ti and Nb, and the widespread interest in ethanol detection, this work aims to comprehend the sensing mechanisms that enhance the performance of one of the most popular semiconductors for gas sensing, namely SnO_2_, by including Ti and Nb in its structure. Then, the study focused on DRIFT spectroscopic investigations of the active sensing elements based on SnO_2_, (Sn,Ti)_x_O_2_ and (Sn,Ti,Nb)_x_O_2_ in real time and under operating conditions, i.e., in the presence of water vapor or ethanol in the testing chamber, with the simultaneous read-out of the sensor activity. The experimental results from *operando* DRIFT spectroscopy led to a better understanding on the surface properties of the metal-oxide solid solution, which could be relevant for fine-tuning of material characteristics for specific applications, e.g., in humid conditions.

## 2. Materials and Methods

### 2.1. Materials Syntheses and Morphological, Structural and Chemical Properties

The materials syntheses, together with their morphological, structural and chemical characterizations, have been extensively presented in previous works [7,24,25,26,27]. Then, this paragraph aims to summarize the key results experimentally demonstrated in [7,24] to provide the readers with the knowledge required to comprehend the discussion on the sensor detection mechanism, assessed using the additional information obtained through the *operando* DRIFT spectrometer.

Nanograined metal-oxide powders were prepared according to sol-gel processes, which started from stoichiometric solutions of Sn (II)-ethylhexanoate, Ti-butoxide and NbCl_5_ in hydro-alcoholic media. The resulting colloids were vacuum-filtered out of the solution, washed multiple times with 2-propanol and water and dried in air at 110 °C for 4 h. The powders were then calcined in air for 2 h in a muffle oven at 650 °C or 850 °C. According to the molar ratio between Sn, Ti and Nb used for the synthesis and to the powder calcination temperature, the samples were labelled as in Table 1.

All the powders were composed of spheroidal nanoparticles with diameters in the range of 5–15 nm for the samples calcined at 650 °C [24,25,27] and between 10 and 55 nm for that calcined at 850 °C [24]. The smaller distribution of STN1.5_650 particle diameters resulted in a larger BET specific surface area and in a remarkably lower average pore size than STN1.5_850. SnO_2__650 exhibited a rutile structure (space group, s.g. *P*4_2_/*mnm*), while ST30_650, STN1.5_650 and STN1.5_850 were mainly composed of a tetragonal rutile-type phase (97–99 wt%, s.g. *P*4_2_/*mnm*), along with a remaining fraction of the tetragonal anatase-type phase (s.g. *I*4_1_/*amd*). By contrasting the unit cell volume of the investigated solid solutions, where 0.0695 < V < 0.0704 nm^3^ [24], with those of pure TiO_2_ (0.0624 nm^3^ [28]) and SnO_2_ (0.0716 nm^3^ [29]) from the literature, it emerged that the rutile-type phase was a solid solution where the smaller ^[6]^Nb^5+^ (ionic radius, i.r. = 0.64 Å) and ^[6]^Ti^4+^ (i.r. = 0.605 Å) replaced about 15% of the larger ^[6]^Sn^4+^ (i.r. = 0.69 Å) at the octahedral site (i.r. from Shannon), leading to a unit-cell volume decrease [24]. Charge compensation after substitution of tetravalent cations, i.e., Sn(IV) or Ti(IV), with pentavalent Nb(V) resulted in Ti(III) defects, revealed through the Electron Paramagnetic Resonance (EPR) and X-ray Photoelectron Spectroscopy (XPS) high-resolution spectrum of the Ti 2p region [7].

### 2.2. Sensor Fabrication

Conductometric chemical thick-film sensors were prepared by using the commercially attractive and low-cost screen-printing process. This method of deposition consists of the production of pastes based on the synthetized nanostructured powders with the addition of alpha-terpineol, ethyl cellulose and ceramic frit, to provide the suitable viscosity for their deposition and to assist the temperature-induced film treatments [30]. Alpha-terpineol (with a mixture of isomers of ≥96%) was from Sigma-Aldrich and was added as an organic vehicle. Ethyl cellulose was from SigmaAldrich (viscosity of a 5% *w*/*w* solution in 80:20 toluene/ethanol with weight at 25 °C) and was used as an organic binder, which increased the shear-thinning pseudoplasticity and the thixotropy of the paste. The organic precursors can be included in a total amount ranging from 50 to 80% of the mass depending on the consistency of the compound to be obtained for an optimal deposition. The ceramic frit consisted of a mixture of glassy oxides based on silica (SiO_2_) charged with alkaline-earth oxides or with oxides of the IV group and constituted a fraction of 0.5%. It had the function of improving the mechanical resistance of the film and its adhesion to the substrate. The pastes with the nanostructured powders and the additives were sonicated in an Elmasonic S 120 H unit to obtain the desired homogeneity.

Such pastes were then printed through a AUREL C920 screen printer with a predefined shape (area of 1.22 × 1.60 mm^2^ and thickness of about 20–30 μm) onto alumina substrates equipped with two interdigitated gold electrodes to supply the input voltage and extract the output signal [31]. Firstly, the film was dried at 150 °C for 4 h to remove the volatile organic compounds. Secondly, a firing at 650 °C for 2 h in a muffle oven completed the decarbonation process and sintering of the semiconducting layer. The two-step process allowed for the limitation of cracks and the production of a uniform and compact film. The substrates were also provided with a platinum screen-printed heater on the backside to set the operating temperature for the sensor thermal-activation. The heater and the interdigitated gold electrodes were interfaced to the electronic system by connecting them with golden wires (diameter of 0.06 mm) to the pins of a commercially available TO39 support through a wedge wire bonder. 

### 2.3. DRIFT Spectroscopy and Electrical Characterization Setup

**Figure 1:**
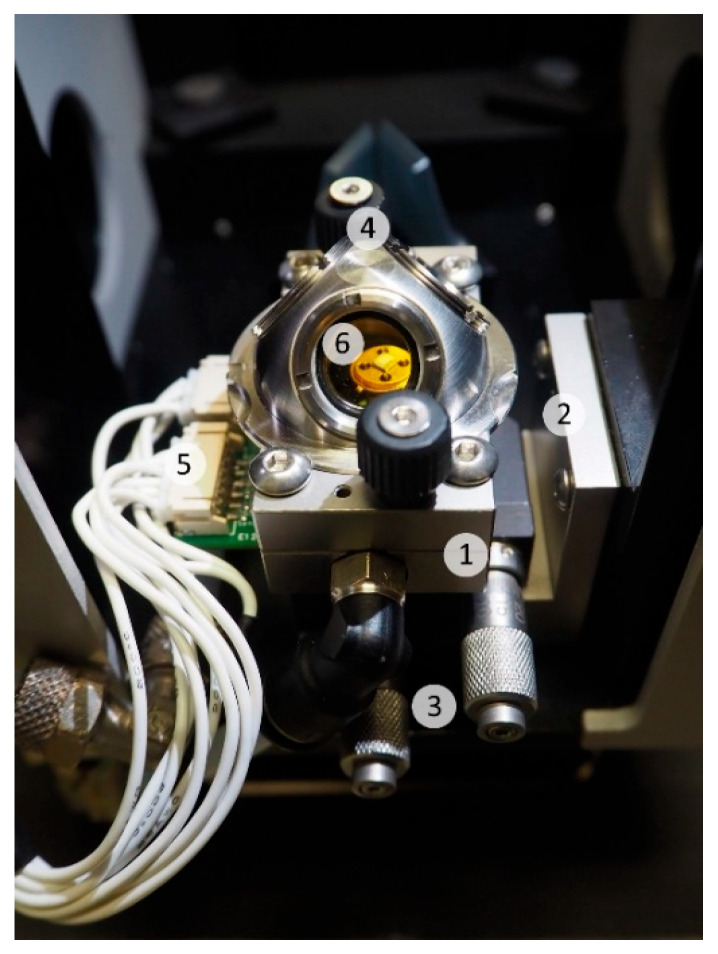
Photo of the operando gas test chamber representing the main cell body (1), the cell support (2), the vacuum-compatible precision XY micro-stage (Standa) (3), the IR dome (4), the connection for electrical measurements via JST connectors (5) and a chemoresistive sensor (6).

The sensing properties of SnO_2__650, ST30_650 and STN sensors were investigated in a customized apparatus for electrical characterizations [32] placed inside the sample compartment of a Vertex 70 IR spectrometer (Bruker) with a liquid-nitrogen-cooled MCT detector used to record the timeresolved DRIFTS spectra [26]. A schematic representation of the setup is shown in Figure S1. The gas sensor was located inside the gas test chamber, together with a commercially available Sensirion SHT3X sensor for temperature and relative humidity monitoring. The gas test chamber, represented in Figure 1, was composed of a main cell body and cell support in 316L stainless steel (SS), a vacuum-compatible precision XY micro-stage (Standa), a dome with two monolithic ZnSe IR windows and a SiO_2_ window for visual alignment (with NIR source) and a connection for electrical measurements via JST connectors.

The flow rates of the dry synthetic air and of the gases coming from certified SAPIO cylinders to the test chamber were set through a pneumatic system based on Brooks SLA5800 mass-flow controllers (error limit of rate equal to 1%). The sensors were kept at their working temperature under a total continuous gaseous flow of 100 sccm (standard cubic centimeters).

The resistance of the sensor was obtained as the ratio of the applied voltage vs. the current. Then, the sensor response (S) was defined as

(1) $$S=\frac{\left(R_{air}-R_{gas}\right)}{R_{gas}}$$

where $R_{air}$ and $R_{gas}$ are the steady state resistance in dry synthetic air and in the presence of the target gas (ethanol or water vapor), respectively.

The gas test chamber was placed inside the sample compartment of an FTIR spectrometer (Bruker, Vertex 70v) and was designed to be compatible with Harrick’s Praying Mantis mirror optics, to work in DRIFT configuration. In order to prevent any absorption interference caused by ambient CO_2_ and water vapor along the beam path, the sample compartment and optics bench were both evacuated to 2 hPa. A vacuum-compatible precision XY micro-stage (Standa), coupled with the Z-axis control system present in the Harrick’s Praying Mantis, allowed the precise alignment of the SMOX film with the IR beam. Operando DRIFT spectra were recorded with a resolution of 4 cm^−1^ and as an average of 600 scans. Bruke’s OPUS software was used to process the IR spectra. To determine the differences in the material resulting from changes in the environmental gaseous composition, absorbance spectra were calculated as apparent absorbance (AB) using Equation (2):

(2) $$AB(\lambda )={-log}_{10}\frac{I_{sample}(\lambda )}{I_{background}(\lambda )}$$

$I_{background}(\lambda )$ and $I_{sample}(\lambda )$ are the intensity of the spectrum (SC) of the sample recorded during exposure to synthetic air and that recorded during exposure to the analyte, respectively.

### 2.4. Experimental Details and Working Conditions

#### 2.4.1. Study under Exposure of Ethanol

The sensors SnO_2__650, ST30_650 and STN were firstly let to stabilize at their best working temperature [18] in the test chamber inside the operando DRIFT spectrometer, under a continuous flux (100 sccm) of dry synthetic air. During ethanol sensing measurements, the flow containing the defined concentration of target gas was obtained by mixing two fluxes, one from a dry synthetic air cylinder and one from a cylinder containing ethanol in a 50 ppm concentration with a relative uncertainty of 2.0% (given from the producer). Equation (3) was used to calculate the flow rate F coming from the ethanol cylinder to be set to obtain a total flow of 100 sccm with an ethanol concentration of 35 ppm. The parameters in Equation (3) are the total flow rate $F_{tot}$ (100 sccm), the ethanol concentration wanted C and the analyte concentration in the cylinder $C_{c}$ (50 ppm). Then, the gaseous composition of the flux was obtained by mixing 30 sccm of dry synthetic air with 70 sccm from the cylinder containing a 50 ppm concentration of ethanol.

(3) $$F=\frac{F_{tot}C}{C_{c}}$$

**Figure 2:**
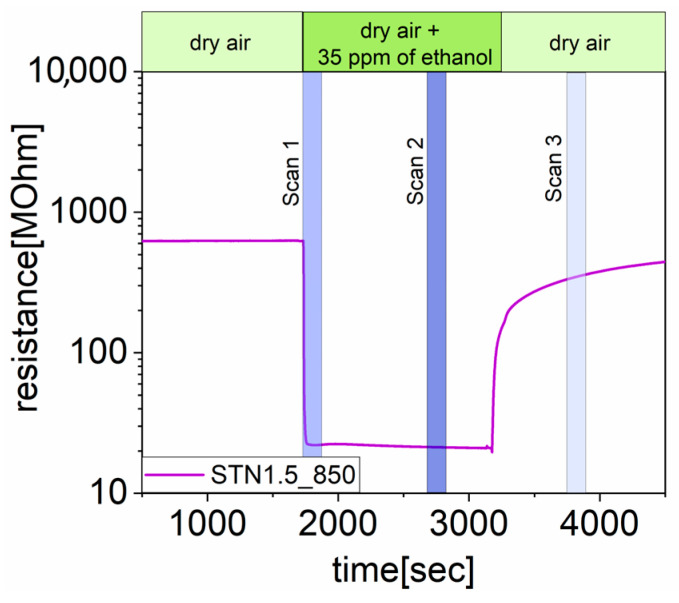
Resistance of STN1.5_850 operated at 450 °C before and after exposure to 35 ppm of ethanol. The green bar shows the gaseous composition of the flow passing through the chamber. The blue bars evidence the periods of time during which the AB spectra were acquired, i.e., scan 1, scan 2 and scan 3 during response time, steady state and recovery time.

To determine the change in the material surface composition resulting from reaction with the analyte, AB spectra were calculated as in Equation (2), in which $I_{background}(\lambda )$ was the spectrum (SC) intensity of the sample recorded during exposure to dry synthetic air. Three AB spectra were collected for each measurement during the response time, steady state condition and recovery time, i.e., the periods of time shown in Figure 2 with colored bars, taken as an explicative example. Contemporarily, the resistance of the sensors was read to quantify their transducer function.

#### 2.4.2. Study in Humid Environment

Water vapor is a significant interference factor that frequently has a negative effect on the chemoresistive device ability to detect the target gases in practical applications. Indeed, the chemical adsorption of H_2_O molecules could directly affect the baseline resistance of the SMOX film while changing the active sites. Moreover, water physiosorbed in continuous layers with the increase in humidity, limiting the access of the analytes to the receptor surface. Therefore, the electrical properties of the SnO_2__650, ST30_650 and STN sensors were tested under different RH% conditions. Humid air was obtained by injecting a fraction of the total 100 sccm synthetic air flux (20% O_2_ and 80% N_2_) into a gas bubbler filled with deionized water. These parameters changed from 1.5 to 45 RH%, by increasing the fraction of total flux passing through the bubbler, and from 35 °C to 39.5 °C, due to the influence of the sensor operating temperature (in the range 350–450 °C) on the small chamber ambient volume (IR dome with a void volume of ≈ 0.5 cm^3^). To determine the change in the material surface composition resulting from reaction with H_2_O, absorbance spectra were calculated as apparent absorbance (AB) using Equation (2), in which $I_{background}(\lambda )$ and $I_{sample}(\lambda )$ were the spectrum (SC) intensity of the sample recorded during exposure to synthetic air and that measured after exposure to humid air, respectively. When a high water vapor content was reached inside the test chamber, the gaseous H_2_O molecules absorbed in large parts of the mid-infrared spectrum (between 4000–3300 cm^−1^ and 2100–1300 cm^−1^), resulting in narrow and intense peaks [33]. This may complicate the analysis of the species adsorbed on the sample. To avoid absorbance peaks of water vapor, AB spectra were obtained in dry conditions after the sensor conductance stabilization in humid air. This method of investigation had the advantage of producing less noisy spectra, but it had the disadvantage of collecting data on a surface that was restarting the cleaning process from sites that had reacted with H_2_O. Therefore, the AB spectrum acquired during exposure to 100 sccm of humid air will also be shown in Section 3.

## 3. Results

Operando DRIFT spectroscopy was performed to deeply investigate the sensing mechanism of SnO_2__650, and the new ST30_650, STN1.5_650 and STN1.5_850 sensors. 

### 3.1. Results under Exposure of Ethanol

**Figure 3:**
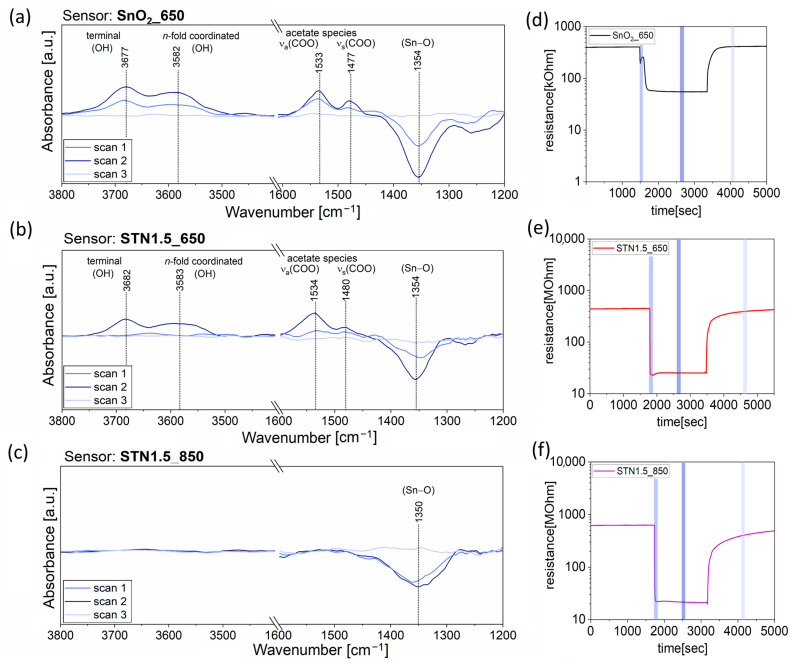
DRIFT AB spectra with background subtraction and signal processing, taken on (**a**) SnO_2__650, (**b**) STN1.5_650 and (**c**) STN1.5_850 during response time (scan 1), steady state (scan 2) and recovery time (scan 3). Graphs (**d**–**f**) show the sensor resistance during the measurements. The blue bars evidence the periods of time during which the AB spectra were acquired. The working temperatures were 400 °C for SnO_2__650 and 450 °C for STN1.5_650 and STN1.5_850. The temperature inside the chamber was in the range of 36–38 °C and RH was around 1.5%.

DRIFT AB spectra were acquired under exposure to 35 ppm of ethanol on SnO_2__650, and on the most promising solid solutions, i.e., STN1.5_650 and STN1.5_850. Figure S2 shows the AB raw spectra in the range 4500–1000 cm^−1^. The AB raw spectra collected during the response time and steady state condition evidenced an increasing baseline from high to low wavenumbers, which has been observed in many previous works from the literature on *operando* DRIFT spectroscopy applied to chemoresistive gas sensors [34,35,36,37] and that can be explained with the increase in charge carriers in the SMOX film after reaction with the analyte [38]. Moreover, they show a raise in characteristic peaks, which have been made more evident in Figure 3 using smooth signal processing and baseline subtraction on OriginPro 2018. On the other hand, the peaks disappeared and the baseline flattened in AB raw spectra acquired during recovery time, due to complete recovery of the surface in dry synthetic air.

DRIFT spectra on SnO_2__650 during exposure to ethanol presented upward peaks at 3677 cm^−1^, 3582 cm^−1^, 1533 cm^−1^ and 1477 cm^−1^ and a downward peak at 1354 cm^−1^ (Figure 3a). Therefore, the reaction produced terminal and *n*-fold coordinated hydroxyl groups (3677 cm^−1^ and 3582 cm^−1^) [35,39,40] while decreasing the content of surface Sn-oxygen (Sn–O) bonds (1354 cm^−1^) [40]. Furthermore, increasing peaks at 1533 cm^−1^ and 1477 cm^−1^ were observed due to asymmetric and symmetric stretch of acetate species (ν_a_(COO) and ν_s_(COO)) formed as intermediates from ethanol reaction [40,41,42]. 

The spectra in Figure 3b revealed that ethanol interaction with the surface of STN1.5_650 resulted in the formation of the species also present on SnO_2__650, namely hydroxyl (3682 cm^−1^ and 3583 cm^−1^) groups and acetate species (1534 cm^−1^ and 1480 cm^−1^). Meanwhile, the decrease in Sn–O bonds was indicated with the downward peak at 1354 cm^−1^. 

On the other hand, the reaction mechanism of ethanol on the surface of STN1.5_850 only decreased Sn–O bond content. Indeed, a single downwards peak was observed at 1350 cm^−1^ in Figure 3c.

Figure 3d,e also evidenced that the sensor based on the three metallic solid solutions displayed a higher response value vs. 35 ppm of ethanol than the pure SnO_2__650, i.e., S = 16.9 for STN1.5_650 and S = 6.3 for SnO_2__650. Moreover, the heating treatment at 850 °C further increased the detection capabilities of STN1.5_850, which reached a response value of 28.9 (Figure 3f).

### 3.2. Results in Humid Environment

**Figure 4:**
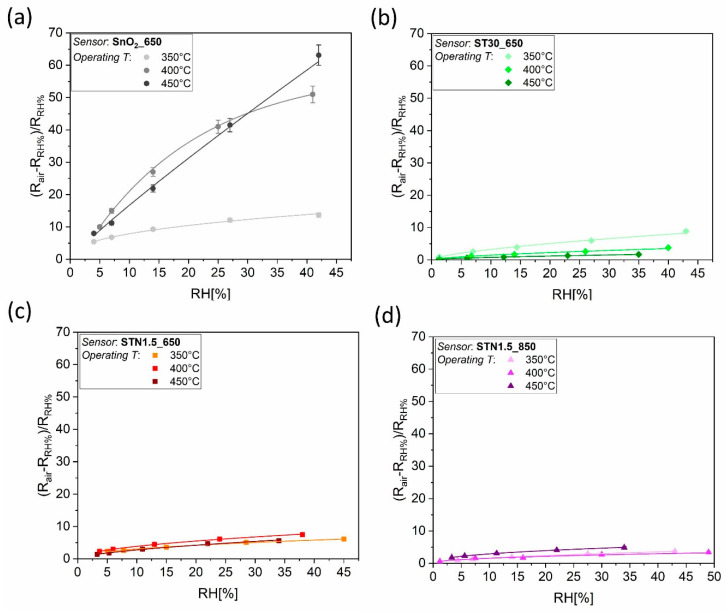
Response level of (**a**) SnO_2__650, (**b**) ST30_650, (**c**) STN1.5_650 and (**d**) STN1.5_850 against all the values of RH% reached inside the chamber, at the three different operating temperatures (350, 400 and 450 °C).

The effect of water vapor on the surface site composition was investigated because, according to previous works [7,24], (Sn,Ti)_x_O_2_ and (Sn,Ti,Nb)_x_O_2_ sensors demonstrated a greater resistance independence from variations of RH% than that based on SnO_2_. Figures S3–S6 collect all the resistance values (resistance vs. time) of the measurements described in Section 2.4.2 for the studies in humid conditions. The sensors were operated at 350 °C, 400 °C and 450 °C in a wide range of RH%. The electrical characterization showed that the resistance of all the sensors decreased as the RH% increased, which is typical of sensors based on n-type materials in the presence of a reducing gas. Figure 4 displays the sensor response against all the values of RH% reached inside the chamber, at the three different operating temperatures. The sensitivity vs. H_2_O of SnO_2__650 operated at 350 °C was magnified when the working temperature of the sensor raised to 400 and 450 °C. On the contrary, the response of ST30_650 decreased by increasing the operating temperature. The behavior of STN1.6_650 and STN1.5_850, instead, varied slightly during all three experiments. By comparing the electrical properties of the four sensors vs. RH% at the optimal temperature for the detection of ethanol [7], i.e., 400 °C for SnO_2__650 and 450 °C for ST30_650, STN1.5_650 and STN1.5_850, it emerged that the resistance of SnO_2__650 was much more affected by humidity than that of Ti-containing sensors. 

**Figure 5:**
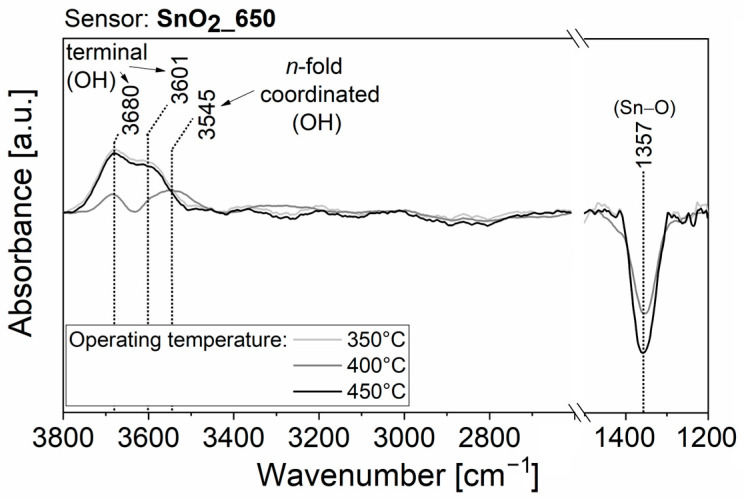
DRIFT AB spectra with background subtraction and signal processing of SnO_2__650 operated at 350 °C, 400 °C and 450 °C and acquired during acquisition periods (5) of Figure S7.

**Figure 6:**
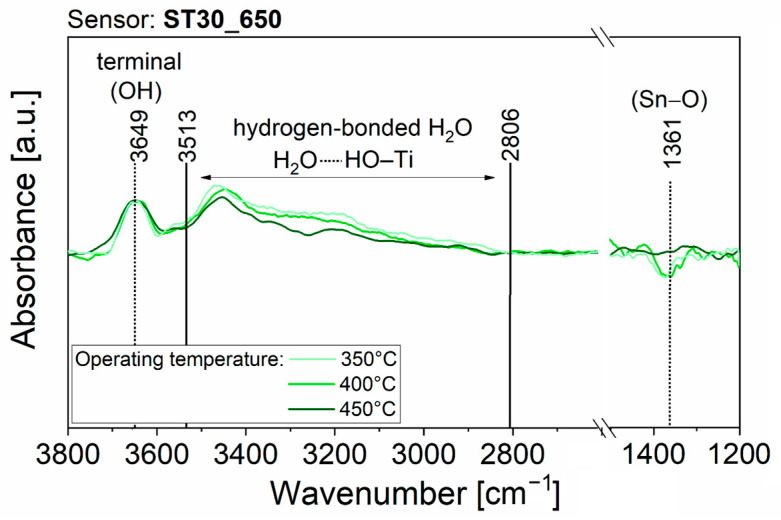
DRIFT AB spectra with background subtraction and signal processing of ST30_650 operated at 350 °C, 400 °C and 450 °C and acquired during acquisition periods (5) of Figure S8.

**Figure 7:**
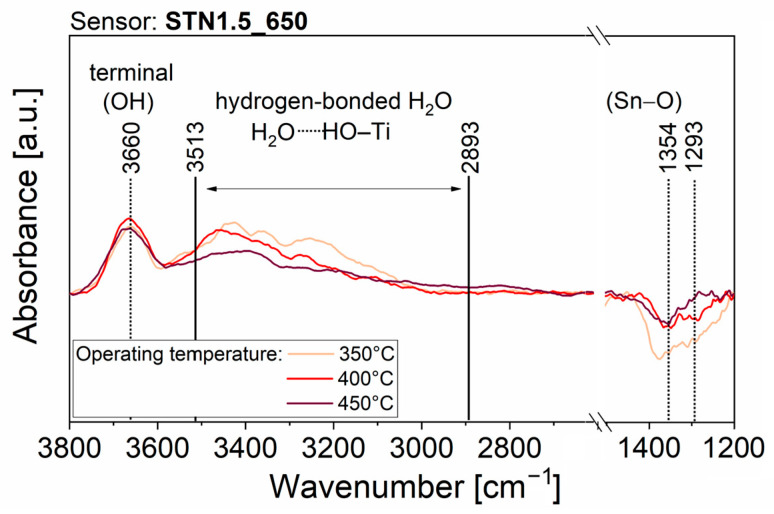
DRIFT AB spectra with background subtraction and signal processing of STN1.5_650 operated at 350 °C, 400 °C and 450 °C and acquired during acquisition periods (5) of Figure S9.

**Figure 8:**
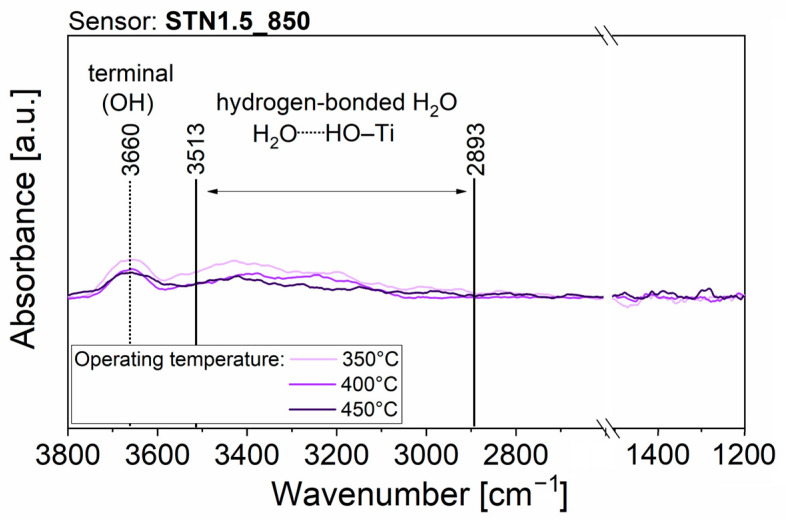
DRIFT AB spectra with background subtraction and signal processing of STN1.5_850 operated at 350 °C, 400 °C and 450 °C and acquired during acquisition periods (5) of Figure S10.

To obtain experimental observations useful for understanding the surface interaction with H_2_O molecules, which causes the resistance variation of the sensing films, AB spectra were acquired during all the previously described electrical measurements, in the time periods indicated with the colored bars in Figures S7–S10. Some features were common across all AB raw spectra. Firstly, after the transition from dry to slightly moist air, the raw spectra showed the appearance of peaks due to surface changes. Secondly, the intensity of the peaks increased with rising RH%, but no new peaks appeared. Thirdly, the AB raw spectra taken during exposure to 100 sccm of humid air (red lines, acquisition (6) in Figures S7–S10) revealed the presence of narrow, and sometimes intense, peaks due to the absorption of gaseous H_2_O molecules in the mid-infrared spectrum (between 4000−3300 cm^−1^ and 2100−1300 cm^−1^). To streamline the discussion regarding the variations that emerged in the spectra obtained on the four films operated at the three different temperatures, only the spectra resulting from the acquisition period (5) of Figures S7–S10 are displayed in Figure 5, Figure 6, Figure 7 and Figure 8. The AB spectra of Figure 5, Figure 6, Figure 7 and Figure 8 were made more evident with smooth signal processing and baseline subtraction on OriginPro 2018. These spectra were chosen to be representative of the results obtained from all the experiments because they were acquired after injection of the highest RH% level.

The AB spectra of SnO_2__650 operated at 350 °C in humid conditions (Figure 5, light grey line) displayed two upward peaks at 3680 cm^−1^ and 3601 cm^−1^ and one downward peak at 1357 cm^−1^. The bands in the wavenumber region between 3000 and 4000 cm^−1^ indicate the formation of new surface hydroxyl groups after exposure to humid air. The band at ~3700 cm^−1^ can be ascribed to isolated terminal hydroxyl groups while that at ~3600 cm^−1^ was in the region of interacting terminal and *n*-fold coordinated hydroxyl groups (between 3600 and 3100 cm^−1^) [35,39,40]. In addition, the absorbance of the band associated with Sn–O overtone [40] at 1357 cm^−1^ decreased, indicating that the formation of hydroxyl groups was accompanied by a decrease in the content of surface Sn-oxygen bonds. Even when operating temperature was raised to 400 °C, the AB spectra after exposure to humid air highlighted an increase in hydroxyl groups and a Sn–O overtone band decrease (Figure 5, grey line). Interestingly, the band at ~3680 cm^−1^ was less intense than that at 3545 cm^−1^, indicating a preference for *n*-fold coordinated hydroxyl group formation. On the other hand, a similar intensity of the bands at 3680 cm^−1^ and 3601 cm^−1^ was observed for SnO_2__650 operated at 450 °C (Figure 5, black line), which suggests that still at a high temperature, different reaction paths were available for H_2_O adsorption.

The AB spectra in Figure 6 (light green line) acquired in humid air on ST30_650 operated at 350 °C show an upward peak at 3649 cm^−1^ and a broad increasing band between 3513 cm^−1^ and 2806 cm^−1^. The peak at 3649 cm^−1^ was attributed to isolated terminal hydroxyl groups. The broad band in the range 3500–2800 cm^−1^ was assigned to water molecules forming a hydrogen-bonded network on the SMOX surface [38,41,43,44]. The AB spectra remained unchanged as the working temperature got higher (see Figure 6, green and dark green lines).

Figure 7 (orange line) highlights an upward peak at 3660 cm^−1^, a broad increasing band between 3513 cm^−1^ and 2893 cm^−1^ and two downward peaks at 1354 and 1293 cm^−1^ in the AB spectra of STN1.5_650 operated at 350 °C, indicating a behavior that partly resembles both that of SnO_2__650 and ST30_650. The new peak at 1293 cm^−1^ is tentatively assigned to Ti–O overtone. Then, as already observed for SnO_2__650, the interaction between water vapor and surface oxygen decreased the number of Sn–O bonds. On the other hand, the upward peak and broad band in the O–H region were ascribed to terminal hydroxyl groups and H_2_O molecules involved in hydrogen bonds, respectively, as for ST30_650. At increasing operating temperatures of 400 °C and 450 °C (Figure 7, red and brown lines, respectively), the changes in the fingerprint region show a lower decrease in surface oxygen species. Indeed, the peaks at 1354 cm^−1^ and 1293 cm^−1^ were far less intense and could not be distinguished from background noise at low humidity (Figure S9). 

DRIFT measurements under exposure to humid air were conducted also on STN1.5_850 to investigate the effect of sintering temperature at 850 °C on the surface reactivity. At a working temperature of 350 °C, the spectrum in Figure 8 (light purple line) shows an upward peak at 3660 cm^−1^ and a broad increasing band between 3513 cm^−1^ and 2893 cm^−1^. The same signals were also present in STN1.5_650 and attributed to terminal OH and hydrogen-bonded H_2_O. Unlike the spectra in Figure 7, already at this temperature no peaks characteristic of Sn–O bond variation were visible. The same was observed in the spectra measured at 400 °C and 450 °C in Figure 8 (purple and dark purple lines), respectively.

## 4. Discussion

As its parent oxides, the solid solution of (Sn,Ti,Nb)_x_O_2_ behaved like an n-type semiconductor material and the film resistance decreased when the sensors were exposed to reducing gases, such as ethanol. Operando DRIFT measurements demonstrated that the sensing mechanism always involved surface oxygen consumption. Indeed, all spectra in Figure 3 show a downward peak at ~1350 cm^−1^ attributed to surface Sn–O bonds [40]. According to the literature [45], oxygen active sites should be mainly $O^{-}$ and $O^{2-}$ over the surface of SMOX films operated between 350 and 450 °C. Under exposure to ethanol at a temperature that provided enough thermal energy for surface reactions, oxygen ions oxidized the chemisorbed target gas molecules and the trapped electrons were released to the conduction band, resulting in a decrease in film resistance. Ethanol can undergo decomposition through a dehydration route (Equation (5)) or a dehydrogenation one (Equation (6)), depending on the acid–base properties of the solid solution. Either ways are followed by consecutive reactions of the intermediate states, which consume the adsorbed oxygens and release free electrons as in Equations (7) and (8) [46,47,48].

(4) $$C_{2}H_{5}OH\to ({C_{2}H_{5}OH)}_{ads}$$

(5) $$({C_{2}H_{5}OH)}_{ads}\to C_{2}H_{4}\;{+H}_{2}O$$

(6) $$({C_{2}H_{5}OH)}_{ads}\to CH_{3}CHO+H_{2}$$

(7) $$({C_{2}H_{4})}_{ads}+{6O^{-}}_{ads}\to 2CO_{2}+2H_{2}O+6e^{-}$$

(8) $$({CH_{3}CHO)}_{ads}+{5O^{-}}_{ads}\to 2CO_{2}+2H_{2}O+5e^{-}$$

**Figure 9:**
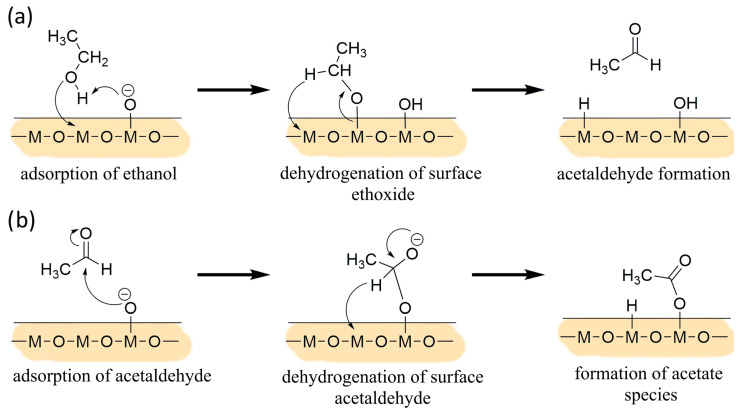
Formation of acetate species from (**a**) dehydrogenation of ethanol and (**b**) consecutive further oxidation of the first reaction product, i.e., acetaldehyde.

AB spectra in Figure 3a,b suggest acetate species formation, which can only be explained with the reaction mechanism in Equation (6) and consecutive further oxidation of the first reaction product, i.e., acetaldehyde [48]. The proposed reaction steps for SnO_2__650 and STN1.5_650 are described in Figure 9. Then, two surface acetate species can proceed through a ketonization reaction to produce acetone and CO_2_ [48,49,50].

A similar conclusion cannot be derived for STN1.5_850 from Figure 3c since AB spectra only revealed a decrease in Sn–O bonds. This evidence implies that ethanol effectively reduced the film surface, but it does not provide any additional information on the sensing mechanism, which was probably too fast to yield observable reaction intermediates. According to these experimental findings, the higher response level of STN1.5_850 than STN1.5_650 should be ascribed to a difference in surface reactivity. Nevertheless, XPS investigations in a preliminary work [7] did not reveal significant differences in surface chemical composition between STN1.5_650 and STN1.5_850. On the other hand, nitrogen gas porosity showed that the average pore size of the sample treated at 850 °C was almost 1.4 times larger than the one calcined at 650 °C (24 nm and 16.7 nm, respectively [7]). Then, the proposed hypothesis is that the larger pore diameters of STN1.5_850 would enhance the Knudsen diffusion of ethanol in the mesoporous film, increasing the production of surface acetate species [7,51]. Consequently, there is a high probability of coupling between two neighboring acetate species to produce acetone. This would result in fast desorption of acetate species, which were not detected in the AB spectrum of STN1.5_850, and in the formation of acetone that could further reduce the material and decrease the film resistance. On the contrary, ethanol molecules would hardly be diffused into STN1.5_650 small pores. This would decrease the probability of coupling between two acetate species resulting from two close ethanol dehydrogenation reactions.

**Figure 10:**
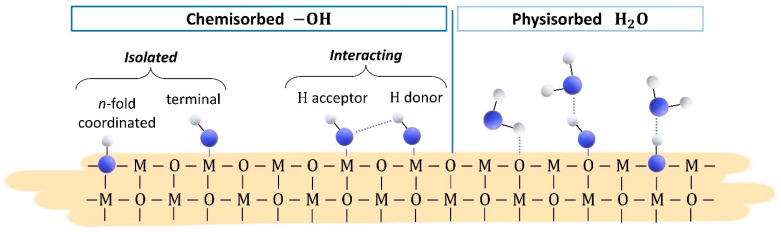
Two-dimensional representation of the metal oxide surface with isolated and interacting hydroxyl groups and physiosorbed water. The blue and white spheres correspond to oxygen and hydrogen atoms, respectively. Solid lines are intramolecular bonds while dotted lines are intermolecular forces, i.e., hydrogen bonds.

Finally, the effect of humidity on the electrical properties of the films was considered because adsorption/desorption processes of H_2_O molecules on the surface may affect the electrical properties of the film as a result of redox reactions. Indeed, electrical characterization of SnO_2__650 in humid air highlighted significant resistance variation correlated with increasing RH%, while AB spectra suggested the formation of new surface hydroxyl groups together with a decrease in Sn–O. The two reaction mechanisms proposed in the literature to explain such observed changes in surface sites of SnO_2_ [40] are represented in Equations (9) and (10). The first mechanism in Equation (9) involves the homolytic dissociation of H_2_O and its reaction with one lattice oxygen, $O_{0}$, to form one terminal hydroxyl group, $\left({Sn}_{Sn}^{\delta^{+}}-OH^{\delta^{-}}\right)$, at a metal M site, $M_{M}$, and one *n*-fold coordinated hydroxyl group, ${\left(OH\right)}_{0}^{+}$, which acts as a surface donor, freeing one electron, $e^{-}$, to the conduction band and increasing sensor conductance. The second mechanism in Equation (10) entails the interaction of H_2_O with lattice oxygen forming two terminal hydroxyl groups, ${Sn}_{Sn}^{\delta^{+}}-OH^{\delta^{-}}$, and one oxygen vacancy, $V_{0}^{++}$. The formation of $V_{0}^{++}$ introduces two electrons into the conduction band, resulting in a relatively larger increase in sensor conductance. Figure 10 schematically represents a metal-oxide surface with isolated and interacting hydroxyl groups formed after H_2_O chemisorption.

(9) $$H_{2}O_{gas}+O_{0}+{Sn}_{Sn}⇆{\left({Sn}_{Sn}^{\delta^{+}}-OH^{\delta^{-}}\right)}_{Sn}+{\left(OH\right)}_{0}^{+}+e^{-}$$

(10) $$H_{2}O_{gas}+O_{0}+{2Sn}_{Sn}⇆2{\left({Sn}_{Sn}^{\delta^{+}}-OH^{\delta^{-}}\right)}_{Sn}+V_{0}^{++}+{2e}^{-}$$

Then, the negligible influence of water to the baseline resistance of ST30_650 and (Sn,Ti,Nb)_x_O_2_ films should be explained. Although DRIFT quantitative analyses are rather complicated, because the signal intensity is sensitive to nanoparticle size, compactness and distribution within the sample [52], the intensity of each peak can be compared to that of the other peaks within the same spectrum. Therefore, qualitative considerations can be drawn from the relative intensities of peaks in spectra from different samples. For example, the intensity of the peak assigned to Sn–O bonds in absorbance spectra of Figure 5 (SnO_2__650 sensor) was equal to or greater than that of the peaks characteristic of O–H bonds. On the contrary, the intensity of the peak assigned to Sn–O bonds in absorbance spectra of Figure 6, Figure 7 and Figure 8 (ST30_650, STN1.5_650 and STN1.5_850 sensors) was smaller or, in the limit case of high operating temperature, not clearly visible compared to the peak and band characteristic of O–H bonds. If the chemisorption mechanisms in Equations (9) and (10) were the only reactions, an increase in O–H peaks should be always followed by a decrease in the Sn–O peak, as in the case of SnO_2__650. This was not observed for ST30_650, STN1.5_650 and STN1.5_850 and much fewer oxygens were involved in the reaction with water. Indeed, most species were molecular H_2_O attached to surface oxygens through hydrogen bonds (Figure 10), whereas the hydroxyls were observed in lesser amounts. This beneficially impacted the electrical stability of the solid solution films in a humid atmosphere, promoting H_2_O non-redox reactions. 

The different adsorption properties of the solid solutions were promoted using substitution of Ti in Sn sites. Already in 1981, Egashira et al. studied the difference of water adsorption properties between SnO_2_ and TiO_2_ [53]. They disclosed that most of the surface oxygens were hydroxylated upon water adsorption on SnO_2_ while on the TiO_2_ surface, most species were molecular water attached to surface oxygens. The reasons behind this experimental evidence were the greater defectivity of SnO_2_, which made its surface more reactive than that of TiO_2_. Density Functional Theory calculations by Bandura et al. also concluded that monolayers of water prefer to be in the molecular form on the TiO_2_ surface while the fraction of the dissociated molecules on the SnO_2_ surface is larger [54]. Although the humidity-independent conductance of TiO_2_ is a significant advantage for gas sensors, the material poor surface reactivity reduces the devic’s sensitivity to most analytes. Then, the substitutional solid solution (Sn,Ti)_x_O_2_ exploited the good sensitivity of SnO_2_ towards most analytes and low cross-sensitivity of TiO_2_ to humidity. On the other hand, the film conductance decreased significantly with the increase in the Ti concentration, going from µS for SnO_2_ to nS for Sn_70_Ti_30_O_2_ in synthetic air under thermal activation. This forces the ST30_650 sensor to operate only at high working temperatures up to 450 °C. Unfortunately, at these operating temperatures, the nanostructured film may suffer from grain coalescence and anatase-to-rutile phase transition [10,55,56]. Then, addition of Nb improved the structural stability of the material, thereby enabling long-term operation at 450 °C [7], i.e., the best operating temperature for low influence of humidity and better sensing performance for STN1.5_650 and STN1.5_850. 

## 5. Conclusions

This work employed *operando* DRIFT spectroscopy on SnO_2_-, (Sn,Ti)_x_O_2_- and (Sn,Ti,Nb)_x_O_2_-based sensors to link the investigations on the transduction function, i.e., the read-out of the device activity, with the investigations on the receptor function, i.e., compositional characterization of the active sensing element in real time and under operating conditions.

AB spectra on SnO_2__650 and STN1.5_650 highlighted the formation of surface acetate species, revealing that the ethanol sensing mechanism involved dehydrogenation to acetaldehyde, which was further oxidized. On the other hand, the AB spectrum of the sensor that best responded to ethanol, i.e., the STN1.5_850, only showed a downward peak due to surface reduction (decrease in Sn–O bonds). The absence of observable reaction intermediates suggested that these quickly led to products that were desorbed from the surface by consuming oxygen. Based on preliminary characterizations [7], it was hypothesized that the coupling of two acetate species was more likely to occur in STN1.5_850 due to its high reactivity and larger pore size, which allowed for better analyte diffusion than in the case of STN1.5_650.

Finally, AB spectra in humid environments demonstrated that Ti promoted non-dissociative adsorption of H_2_O through hydrogen bonds rather than redox reactions and consequently hydroxyl group formation. This gain was maintained even in the solid solution doped with Nb. The negligible influence of water vapor on the conductance of the (Sn,Ti,Nb)_x_O_2_ films is a significant advantage because the effect of ambient humidity on the baseline and sensitivity often reduces sensor accuracy and complicates calibrations. 

## Figures and Tables

**Table 1:** Sensor labels according to the molar ratio between Sn, Ti and Nb used for the synthesis and the calcination temperature.

Sensor Label	Sn:Ti:Nb Molar Ratio	Calcination Temperature
SnO_2__650	100:0:0	650 °C
ST30_650	70:30:0	650 °C
STN1.5_650	69.0:29.5:1.5	650 °C
STN1.5_850	69.0:29.5:1.5	850 °C

## Data Availability

Data available on request. The data presented in this study are available on request from the corresponding author.

## Supplementary Materials

The following supporting information can be downloaded at: https://www.mdpi.com/article/10.3390/nano13192708/s1, Table S1: The ethanol-sensing performance of metal oxide sensors in the literature; Figure S1: Schematic representation of operando DRIFT experimental setup; Figure S2. AB raw spectra taken during the measurements with 35 ppm of ethanol; Figure S3. SnO_2__650 baseline resistance variation under exposure to different RH%; Figure S4. ST30_650 baseline resistance variation under exposure to different RH%; Figure S5. STN1.5_650 baseline resistance variation under exposure to different RH%; Figure S6. STN1.5_650 baseline resistance variation under exposure to different RH%; Figure S7. Evolution of the DRIFT AB spectrum during the increase of RH% on the SnO_2__650 sensor; Figure S8. Evolution of the DRIFT AB spectrum during the increase of RH% on the ST30_650 sensor; Figure S9. Evolution of the DRIFT AB spectrum during the increase of RH% on the STN1.5_650 sensor; Figure S10. Evolution of the DRIFT AB spectrum during the increase of RH% on the STN1.5_850 sensor. References [57,58] are cited in the Supplementary Materials.
